# On the Three Images Reflected in the Eye

**Published:** 1844-06-15

**Authors:** 


ON THE THREE IMAGES REFLECTED IN THE EYE.

M. Magne lately addressed a note to the Academy of Sciences, relative to the three images reflected in the eye by the light of a taper.

The late Professor Sanson, began to observe in 1836, and pointed out to his clinical class in 1837, that when a candle is placed before the eye of an amaurotic patient, whose pupil is dilated, three images of the flame can always be distinguished, succeeding one another from before, backwards. The first, or anterior one, which is the brightest, is erect; the second, or middle one, which is less bright, is reversed; and the third, or posterior one, is much fainter than the other two, and is erect, like the first one. M. Sanson and his pupils, arrived at the same results, and ascertained that the anterior erect image, is produced by the cornea; that the middle reversed one, is owing to the posterior segment of the crystalline capsule; while the posterior erect image arises from the anterior segment of the same capsule. Opacity of the cornea destroys all three images. Opacity of the anterior capsule destroys the two posterior images. Opacity of the posterior capsule prevents the production of the reversed image.

In other words, when there is a posterior capsular cataract, we cannot see the middle or reversed image; in anterior capsular cataract, the anterior erect image is alone visible; and so it is in capsulo-lenticular cataract. The experiments of M. Pasquet, combined with these, have confirmed the conclusion that a cataract, even at its commencement, can always be distinguished from amaurosis and glaucoma. For since in glaucoma and amaurosis, the alteration does not affect the crystalline apparatus, the three images of the flame still remain.—*Lond. Med. Gaz. from Gazette Médicale*, Jan. 27, 1844.

